# Melanoma in the elderly patient: a single institution retrospective analysis

**DOI:** 10.1186/1471-2482-13-S1-A16

**Published:** 2013-09-16

**Authors:** Vincenzo Desiato, Gennaro Quarto, Stefano Perrotta, Gian Luca Benassai, Bruno Amato, Giacomo Benassai

**Affiliations:** 1 University Department of General Surgery, Geriatric, Oncologic and Advanced Technologies, Faculty of Medicine and Surgery, Federico II University Hospital, Via S. Pansini 5, 80131, Napoli, Italy

## Background

Debate around the reliability of age as a prognostic factor in melanoma patient has been intense. Age is likely a prognosis affecting factor in melanoma, either directly or indirectly, as it causes differences in diagnosis, biologic behavior and therapy. Furthermore there is lack of data from widest randomized controlled trials, because of the exclusions criteria for the elderly. Therefore the management of elderly patients with melanoma remains challenging. We undertook this study to identify factors associated with outcome.

## Methods

A retrospective analysis was carried out on elderly (age ≥ 65) melanoma patients treated in our institution from January 1995 to January 2008. We identified and analyzed 77 patients. A minimum of 60 months follow-up was available on all surviving patients. Mean follow up was 50.86 ±35.7 months. A detailed retrospective analysis was conducted to identify prognostic factors for Disease Free Survival (DFS) and for Overall Survival (OS).

## Results

The mean age was 76.26 ±7.07 years. Forty-two patients (55%) were male. Primary lesion sites were on lower limb (41.6%), trunck (35%), upper limb (14.3%), head or neck (9.1%). Mean Breslow thickness was 7.8 ±12.92 mm. Mitotic rate (mitoses/mm^2^) was divided into four groups: < 1 mitosis 14.3%, 1 ≤ mitoses < 3 35%, 3 ≤ mitoses < 5 19.5%, ≥ 5 mitoses 31.2%. Ulceration was present in 54.5% of cases. There was a prevalence of nodular melanoma (52%), followed by superficial spreading (35%), lentigo maligna (9.1%) and acral lentiginous (3.9%). All patients were adequately treated considering margins and lymphatic basins management. Almost all underwent sentinel lymph node biopsy (SLNB), except two cases of thin melanoma (0.5 and 0.7 mm of thickness). We observed positive SLNB in 16/75 cases (21.3%) with following completion lymph node dissection (CLND) that classified as N1 7 patients, as N2 6 patients and as N3 3 patients. Three patients (3.9%) underwent total lymph node dissection (TLND) for clinically positive lymph nodes. Kaplan-Meier analyses were performed to determine the overall survival, 5-year DFS and 5-year OS for different risk factors. All statistical analyses used SPSS version 19. With α-criterion fixed at 0.05 we observed, in our cohort, that lymph node status, primary tumor thickness, mitotic rate and gender are very important prognostic factors in the elderly patient with melanoma.

## Conclusions

As for the younger patient, aforementioned prognostic factors affect the prognosis in the elderly. Age, for its part, may worsen prognosis, increasing recurrence rates and negatively affecting DFS and OS. That being said, we should treat elderly patients as the younger and, since often the general conditions of aged patient preclude more aggressive medical therapies, surgery remains the most important therapeutic tool. Furthermore, as we know, the great challenge of our day is the early diagnosis.
